# Chemical Composition, Insecticidal, Persistence and Detoxification Enzyme Inhibition Activities of Essential Oil of *Artemisia maritima* against the Pulse Beetle

**DOI:** 10.3390/molecules27051547

**Published:** 2022-02-25

**Authors:** Nandita Chauhan, Urvashi Kashyap, Shudh Kirti Dolma, Sajjalavarahalli G. Eswara Reddy

**Affiliations:** 1 Entomology Laboratory, Agrotechnology Division, CSIR-Institute of Himalayan Bioresource Technology, Palampur 176061, India; nanditachauhan796@gmail.com (N.C.); kashurvi5991@gmail.com (U.K.); skdolma@gmail.com (S.K.D.); 2 Academy of Scientific and Innovative Research (AcSIR), Ghaziabad 201002, India

**Keywords:** fumigant, persistence, repellence, ovipositional, AChE, GST

## Abstract

Pulse beetle is the major pests of pulses that cause significant loss to grains leads to unfit for consumption and marketing. Indiscriminate use of synthetic pesticides for the control of pulse beetle (*Callosobruchus chinensis* and *Callosobruchus maculatus*) led to insect resistance, pesticide residues on grains which affect consumer’s health and environment. Essential oils (EOs) are good alternatives to synthetics due to their safety to the environment and consumers’ health. The main objective of the present study was to explore the chemical composition, fumigant, repellency, ovipositional deterrence, persistence, and detoxification enzyme inhibition of *Artemisia maritima* essential oil against pulse beetle. Results showed that primary components of the EO were 1,8-Cineole and bornyl acetate. EO showed promising fumigant toxicity to *C. chinensis* and *C. maculatus* (LC_50_ = 1.17 and 0.56 mg/L, respectively) after 48 h. In the repellent assay, EO at 8 mg/L showed 92–96% repellence after 1 h. In ovipositional deterrence assay, EO showed more ovipositional deterrence against *C. chinensis* (OD_50_ = 3.30 mg/L) than *C. maculatus* (OD_50_ = 4.01 mg/L). Higher concentrations of oil (8 and 6 mg/L) in *C. maculatus* showed significant inhibition of the glutathione-S-transferase enzyme (7.14 and 5.61 n mol/min/mL, respectively).

## 1. Introduction

Infestation of bruchids and lepidopterans pests causes significant damage to grains and their products in storage. More than 500 species of the stored grains and cereal products are often infested by more than 600 species of coleopterans [1] causing 20–30% loss, affecting nutritional value and germination [2]. Bruchids, *Callosobruchus chinensis* and *C. maculatus* (Coleoptera: Bruchidae), are primary pests of pulses and cause 50% loss in storage after three to four months [3,4]. The grub’s bore into grains, feed internal contents, affecting nutritional quality [5]. In severe infestation, seeds become completely hollow and unsuitable for marketing [6]. The control of stored grain pests generally depends on synthetic insecticides, including fumigants [5,7]. The use of synthetic insecticides resulted in several negative effects in the environment, natural enemies, human health, and resistance development in insects. Now the focus is shifted to search of potent insecticide from natural origin. Plant essential oils (EOs), its monoterpenoids and sesquiterpenoids are known to possess significant insecticidal activities against stored grain pests [8,9,10] The EOs are extracted from different parts (leaves/flowers/seeds/bark) of aromatic and medicinal plants. Nearly 10% of EOs used in aromatic, flavor and fragrance industries [11].

Sea wormwood (*Artemisia maritima* L.) is an aromatic perennial herb distributed in the western Himalayas (Kashmir, Himachal Pradesh, and Uttarakhand [12]. Essential oil is used for antibacterial, antifungal, antispasmodic, antimalarial, and bronchodilatory activities [12,13,14]. Presently, EOs and their secondary metabolites of *Artemisia* spp. are extensively used in folk/modern medicine, cosmetics, food, forage, and pharmaceuticals for the control of malaria, hepatitis, cancer, inflammation, and infections caused by fungi/bacteria/viruses [14,15,16,17] and treatment of COVID-19 [18]. The EO from *A. annua*, *A. judaica*, *A. dracunculus*, *A. santonicum*, *A. spicigera*, *A. vulgaris*, *A. scoparia,* and *A. sieberi* showed contact, fumigant, repellent, and ovipositional activities against pulse beetle [19,20,21,22,23,24] and other pests [16,25,26]. Insecticidal activities of *A. maritima* oil are reported in some insect pests but no report on pulse beetle. In this study, the main objective of the investigation was to evaluate the *A. maritima* oil for fumigant, repellent, ovipositional, persistent activity, and detoxification enzyme inhibition against pulse beetle.

## 2. Results

### 2.1. Chemical Composition of A. maritima Oil

A total of 14 compounds-accounting for 98.51% of *A. maritima* were identified by GC and GC-MS. The oil was composed of 28.11% monoterpene hydrocarbons, 45.73% oxygenated monoterpene fraction, and 2.42% oxygenated sesquiterpene. The major components of the oil were 1, 8-cineole (41%) and bornyl acetate (18.10%) followed by myrcene (9.59%), sabinene (6.42%), camphene (3.74%), and β-phellandrene (3.68%). Other components included terpinolene, germacrene-D, santolina triene, chrysanthenyl acetate (Table 1).

### 2.2. Fumigant and Persistence Toxicity of A. maritima Oil against Pulse Beetle

Fumigant toxicity and persistence of *A. maritima* oil against pulse beetle were presented in Table 2, Table 3 and Table 4. The oil showed more promising toxicity against *C. maculatus* (LC_50_ = 1.91 and 0.56 mg/L) after 24, and 48 h of treatment respectively as compared to *C. chinensis* (LC_50_ = 2.06 and 1.17 mg/L) (Table 2). Based on persistence study, *A*. *maritima* oil showed significantly more promising in protecting the grains/seeds (Table 3) against *C. chinensis* (82% mortality) up to 10 days of storage (F_3,19_ = 55.30; *p* < 0.0001) as compared to *C. maculatus* (44% mortality) (F_3,19_ = 33.47; *p* < 0.0001). *A. maritima* oil showed moderate residual toxicity of 10–20 days only, later toxicity was gradually decreased and became 8 and 0% mortality, against *C. chinensis* and *C. maculatus*, respectively after 40 days of storage. *A. maritima* oil also showed residual toxicity with the lethal time taken to kill 50% of test insects was 4.49 days and 9.33 days for *C. chinensis* and *C. maculatus*, respectively (Table 4). The seeds used for the persistence study also showed 100% germination within 24 h.

### 2.3. Repellency of A. maritima Oil against Pulse Beetle

Repellency of *A. maritima* oil against *C. chinensis* after 1, 2, 3, 4, and 5 h of after treatment was presented in Table 5. Higher concentration (8 mg/L) of *A. maritima* oil showed significantly higher repellence (92%) after 1 h (F_4,24_ = 11.97; *p* < 0.0001) and remain effective up to 5 h (88% repellence) and was at par with 6 mg/L (80% repellence) as compared to lower concentrations (12–36% repellence). Similarly, *A. maritima* oil at 8 mg/L showed significantly higher repellence (96%) against *C. maculatus* after 1 h (F_4,24_ = 10.61; *p* < 0.0001) and was at par with 6 mg/L (76% repellence) as compared to lower concentrations (28–56% repellence). Based on the repellent index (Table S1), all the concentrations of the *A. maritima* oil showed indifferent (I).

### 2.4. Ovipositional Deterrence of A. maritima Oil against Pulse Beetle

Results on ovipositional deterrence of *A. maritima* oil against *C. chinensis* and *C. maculatus* was presented in Table 6 and Tables S2 and S3. *A. maritima* oil was showed promising deterrence against *C. chinensis* (OD_50_ = 2.3, 3.09 and 3.30 mg/L) after 24, 48, and 72 h of treatment, respectively (Table 6) as compared to *C. maculatus* (OD_50_ = 2.89, 3.36 and 4.01 mg/L). With respect to percent deterrence against *C. chinensis*, the *A. maritima* oil at higher concentration (12 mg/L) reported 100% deterrence against *C. chinensis* at 24 h of treatment (F_4,24_ = 16.08; *p* < 0.0001) and was at par with other concentrations (78.2 to 94.38% deterrence) except 2 and 1 mg/L. Similarly, 48 and 72 h after treatment, *A. maritima* oil at 12 mg/L showed higher deterrence (98.28–100%) and was at par with 8 mg/L (92.34 to 93.28% deterrence) and was followed by 4 and 2 mg/L which were at par as compared to lower concentration. Similarly, for *C. maculatus*, *A. maritima* oil at 8 mg/L showed 100% deterrence after 24 h against *C. maculatus* (F_4,24_ = 30.38; *p* < 0.0001) and was at par with 4 mg/L (86.54%) followed by 2 mg/L (70%) as compared to lower concentrations. At 48 h after treatment same trend was observed as that of 24 h. At 72 h, *A. maritima* oil at 8 mg/L showed significantly higher deterrence (81.78%) (F_4,24_ = 41.78; *p* < 0.0001) and was at par with all other concentrations except lower concentration.

### 2.5. Detoxification Enzyme Inhibition of A. maritima Oil against Pulse Beetle

**Figure 1:**
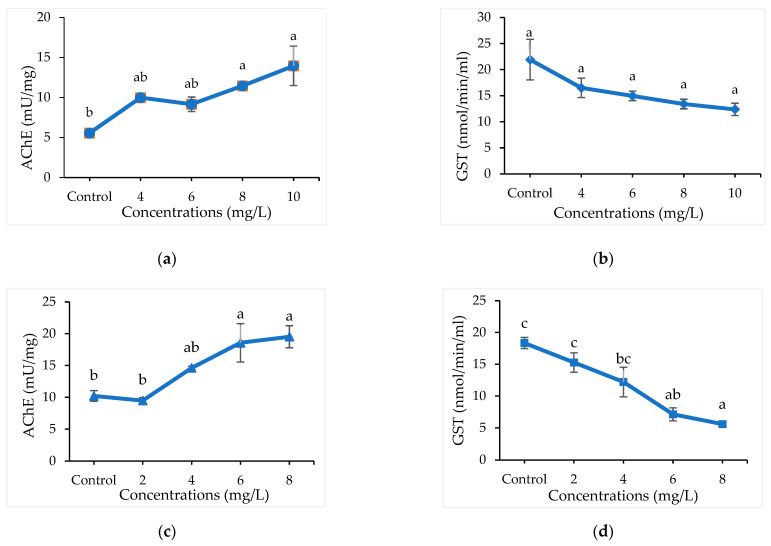
Detoxification enzyme inhibition activities: AChE (**a**) and GST (**b**) in *C. chinensis*; AChE (**c**) and GST (**d**) in *C. maculatus* treated with *A. maritima* oil; Bars represents a standard error (±SE) of three replications; Means followed by the same letters within a figure do not differ significantly by Tukey’s HSD (*p* ≤ 0.05).

Detoxifying enzyme (AChE and GST) activities of *A. maritima* oil against *C. chinensis* and *C. maculatus* after 12 h of treatment is presented (Table S4 and Figure 1). Significant differences were not observed (*p* > 0.001) among the concentrations of oil (4 to 10 mg/L) in inhibiting the enzyme GST and AChE in *C. chinensis*. Similarly, higher concentrations of *A. maritima* oil at 6 and 8 mg/L significantly inhibited the GST (7.14 and 5.61 n mol/min/mL) (F_4,14_ = 14.6; *p* < 0.003) and AChE (mU/mg) (F_4,14_ = 8.14; *p* < 0.003) in *C. maculatus* and were at par as compared to lower concentrations and control.

## 3. Discussion

The chemical composition, insecticidal, and enzyme inhibition activities of *A. maritima* oil against *C. chinensis* and *C.* *maculatus* are discussed. Present results revealed that *A. maritima* oil is rich in oxygenated monoterpenes (45.73%) and monoterpene hydrocarbons (28.11%). The major constituents of the oil were 1,8-cineole, bornyl acetate, myrcene, and sabinene which account for more than 75.25% of the total oil. The earlier studies on Artemisia species also reported 1,8-cineole was the major compound (9.91, 12.96, and 19.59%) in the EO of *A. sieberi*, *A. gmelinii*, and *A. herba alba*, respectively [27,28,29] but lesser than the present study (41.14%). Similarly, 1,8-cineole present in *A.* *maritima* oil (23.6–25%) was also lesser [30,31,32] than in the present study. The β-myrcene (5.09–5.83%) in *A. campestris* and *A. absinthium* was also lesser [27,33] than present study (9.59%). The variation in the chemical constituents might be due to environmental conditions (climate, season, and geographical variation), location/altitude, stage of the plant, time of collection, species/chemotype, and nutritional status of the plant [34,35,36].

EO of different species of Artemisia showed biological activity to pests. As per the information, the current report is the earliest to disclose that the essential oil of *A. maritima* showed promising results in toxicity, repellent, and ovipositional deterrence against *C. chinensis* and *C. maculatus* adults. The previous studies also reported that the EO from other Artemisia species including *A. annua* [19], *A. judaica* [20], *A. monosperma* [37], *A. dracunculus*, *A. santonicum*, *A. spicigera* [21], *A. herba-alba*, *A. campestris*, and *A. absinthium* [27], *A. ordosica* [38], *A. vulgaris* [22], *A. scoparia* [23], *A. sieberi* [24] and *A. annua* [39] showed contact, fumigant, repellent, and ovipositional activities against pulse beetle. Insecticidal activities of the EO depend upon the major constituents, concentration, application method, stage, and type of insect. [28,40,41] In the present study, our results revealed that *A. maritima* oil showed fumigant toxicity against *C. chinensis* (LC_50_ = 1.17–2.06 mg/L) and *C. maculatus* (0.56–1.91 mg/L) within 24–48 h as compared to *A. herba alba*, *A. albsinthium* and *A. campestris* (LC_50_ = 8.3–30.5 μL/L air) against *Bruchus rufimanus* [27]. In a related study, *A. dracunculus*, *A. santonicum*, and *A. spicigera* (5 μL/L air) showed 88–95% mortality [21] and *A. sieberi* showed promising fumigant toxicity (LC_50_ = 1.45 μL/L air) against *C. maculutus* [24]. Similarly, the EOs of *Mentha spicata*, *M. piperita*, and *Tagetes minuta* showed promising fumigant toxicity against adults of *C. chinensis* (LC_50_ = 0.9–1.4 µL/mL) and *C. maculatus* (LC_50_ = 1.1 to 2.0 µL/mL) [4].

In the present study, the EO of *A. maritima* showed 84–96% repellence and ovipositional deterrence (OD_50_ = 3.30–4.01 mg/L) against *C. chinensis* and *C. maculatus*. The present results are similar to the earlier studies, where EOs of *M. spicata*, *M. piperita*, and *T. minuta* showed 84 to 96% repellency [4]. In another study, the EO of *Ocimum gratissimum* exhibited 73–93% repellence against *C. chinensis* after 24 h [42]. Similarly, *M. spicata* and *M. piperita* oil at 12 µL/mL also showed 100% ovipositional inhibition against *C. chinensis* and *C. maculatus* [4]. The EOs of *Lippia alba* Mill. and *Callistemon lanceolatus* (Curtis) at 100 µL/L [43] reported 66–96% oviposition deterrent against pulse beetle; whereas the EOs of *Illicium verum* and *Croton anisatum* at 17.5 µL/L [44] also showed 100% oviposition deterrent against *C. chinensis* as compared to the present study.

The insecticidal activities of *A. maritima* oil against targeted insects in the present study may be due to the presence of major compounds including 1,8-cineole, bornyl acetate, myrcene, and sabinene. Current results also confirmed with the earlier studies in which insecticidal activities of oils are due to monoterpenoids and sesquiterpenoids which are volatile and rather lipophilic compounds that can penetrate insect cuticle and interfere with their physiological functions [45,46,47]. Du to th volatile nature of EOs, they act as a fumigant and kill the stored-grain insects by asphyxiation. The insecticidal activities also depend on nature and type of components, application dose/concentrations [48,49,50].

Normally, insects utilize detoxification enzymes to metabolize xenobiotics [51,52,53]. However, enzymes can be induced by botanical and chemical insecticides which play a significant role in developing resistance to pests [54,55]. GST enzyme involved in detoxification of insecticides of organophosphate, organochlorines, pyrethroids, carbamates, etc., [56,57]. The chemical constituents present in the EOs and their blends, inhibit the GST activity [58,59,60]. In the current studies, *A. maritima* oil was not significantly inhibiting GST/AChE enzyme in *C*. *chinensis* and AChE in *C. maculatus*. However, higher concentrations of *A. maritima* oil showed inhibition of GST enzyme in *C. maculatus* and these results confirm the findings of previous work in which *A. brachyloba* oil significantly inhibited the GST in *T. castaneum* after 24 h but the same oil also inhibit the AChE after 60 h of treatment [61]. In a similar study, eucalyptol and caryophyllene oxide isolated from EO of *A. lavandulaefolia* showed inhibition of GST and CarE in the larvae of *Plutella xylostella* after 24 h [26]. Based on insecticidal activities, *A. maritima* oil showed promising fumigant toxicity, repellence, ovipositional deterrence, and enzyme inhibition (GST and AChE) activities against *C. chinensis* and *C. maculatus*.

## 4. Materials and Methods

### 4.1. Plant Material

The plant material was collected from Keylong (Lahaul & Spiti District, H.P, India) (latitude 32.571° N and longitude 77.041° E) of Himachal Pradesh during September 2019. The specimens are authenticated by the Taxonomist, and a voucher specimen (PLP 17794) was deposited in the herbarium.

### 4.2. Extraction of A. maritima Oil

The whole plant (aerial parts) material of *A. maritima* was dried under shade for 10 days and chopped into small pieces. About 10 kg plant material was used for extraction of oil by hydro-distillation in Clevenger apparatus as per the method followed [16]. The yield obtained was 3.4 mL and was kept below 4 °C until further use.

### 4.3. Gas Chromatography Analysis

The composition of EO determined by gas chromatography (GC) on a Shimadzu GC 2010 equipped with DB–5 (J & W Scientific, Folsom, CA, USA) fused silica capillary column (30 m × 0.25 mm i.d., 0.25 µm film thickness) with a flame ionization detector (FID) [16,62]. The GC oven temperature programmed at 70 °C (initial temperature) held for 4 min and then increased at a rate of 4 °C/min to 220 °C and held for 5 min. The injector temperature was 240 °C, the detector temperature, 260 °C, and the samples were injected in split mode. The carrier gas was nitrogen at a column flow rate of 1.05 mL/min (100 kPa). The sample’s retention indices (RI) were determined based on homologous *n*-alkane hydrocarbons under the same conditions.

### 4.4. Test Insect

*C. chinensis* and *C. maculatus* were maintained on green gram seeds in plastic jars under controlled conditions (27–28 °C and 60 ± 5% humidity) in the Entomology laboratory, Agrotechnology Division, CSIR-IHBT, Palampur for >50 generations. The newly emerged adults (2–3 days old) were used for the study.

### 4.5. Fumigant Toxicity of A. maritima Oil against Pulse Beetle

Five different concentrations of *A. maritima* oil for *C. chinensis* (10, 8, 6, 4, and 2 mg/L) and *C. maculatus* (8, 4, 2, 1, and 0.5 mg/L) were prepared based on preliminary evaluation for dose–response bioassay against pulse beetle adults. The fumigant toxicity assay was studied on glass desiccators (2.5 L capacity). Five grams of green gram were taken in a glass Petri dish and kept at the bottom of the desiccators in which 10 adults are released. In another Petri dish, Whatman No. 9 filter paper was placed and kept at the top of the desiccators. Five concentrations of *A. maritima* oil were applied on the filter paper separately by using a micropipette and then the lid of the desiccators was closed to make it airtight. The desiccators were kept in the controlled laboratory conditions for recording the mortality at 24 h intervals. There are five treatments/concentrations, and each treatment was replicated thrice.

### 4.6. Persistence of A. maritima Oil against Pulse Beetle

The persistence of *A. maritima* oil was carried out as per the standard method followed by Nenaah [8]. Higher concentrations (4 and 8 mg/L) of oil were used against *C. chinensis* and *C. maculatus*, respectively. Briefly, sterilized green gram seeds were treated with two concentrations of EO and seeds were vigorously hand-shaken for 20–30 s for thorough coating. After evaporation of the solvent, the treated grains were packed in jute sacks (20–30 cm) and stored in dark conditions. Samples of treated seeds (20 g) were withdrawn after 10, 20, 30, and 40 days of treatment and then 10 adults (1 day old) are released in the Petri-dish (9 cm diameter). The same no. was also used for the control treated with 0.05% of Triton-X 100 LR water. Each treatment was replicated five times. The insects were exposed to treated seeds were continued for 48 h and then mortality was recorded. For germination study, the treated green gram seeds with *A. maritima* oil were placed on moistened cotton in a Petri dish and incubated under laboratory conditions. Observations on the number of seeds germinated were recorded after 24 and 48 h.

### 4.7. Repellent Activity of A. maritima Oil against Pulse Beetle

Repellent activity of *A. maritima* oil was tested against *C. chinensis* and *C. maculatus* as per the method followed by Eccles et al. [63]. Briefly, five concentrations (8, 6, 4, 2, 1 mg/L) were prepared from stock solutions. The Whatman No. 9 filter paper (diameter 9 cm) was cut and marked with a pencil into two halves and each labeled as treated (T) and untreated (UT). Filter papers were transferred to Petri plates (diameter 9 cm) and treated with required concentrations of EOs and then allowed to air dry for 15 min. Ten adults (3–4 days old) were released in the center of the filter paper containing ten grains, and the plates were sealed with parafilm to prevent the escape of adults. The dispersal of the beetles on each side of the filter paper was recorded after treatment. Observation on repellency was recorded at 1, 2, 3, 4, and 5 h after treatment. In this study, there were five treatments, and each treatment was replicated five times. About 250 insects were used in different treatments (5 treatments × 50 insects = 250 insects). The Percent repellency (PR) [64] was calculated based on the formula: PR = [(Nc − Nt)/(Nc + Nt)] × 100. Where Nc = number of insects on control half of filter paper after required exposure interval; Nt = number of insects on treated half of filter paper after required exposure interval.

The Repellent Index (RI) [65] was calculated based on the formula; RI = 2G/G+P. Where G = number of adults on the treated side and P = number of adults on the untreated side. The repellent index of EOs is considered as repellent, attractant, or indifferent based on the mean value of RI and its respective standard deviation (SD). If the mean RI is higher than 1 + SD, the oil is an attractant, while if the mean RI is less than 1-SD, the oil is repellent, and for the mean RI in between 1 − SD and 1 + SD, the oil is indifferent.

### 4.8. Ovipositional Deterrent Activity of A. maritima Oil against C. chinensis and C. maculatus

The ovipositional deterrent of *A. maritima* oil against *C. chinensis* and *C. maculatus* was studied as per the method followed by Eccles et al. [63]. Briefly, five concentrations (12, 8, 4, 2, 1 mg/L) for *C. chinensis* and *C. maculatus* (8, 6, 4, 2, 1 mg/L) were prepared from stock solutions by mixing EOs in acetone. Seeds (30 no./plate) dipped in different concentrations for 10s, then removed and placed on filter paper to air dry for 15 min. Treated seeds were placed in a Petri plate (diameter 9 cm) and then ten adults (5 male and 5 female) of one day old were released. Petri plates were sealed with parafilm to prevent the escape of the adults. For the control, seeds were treated with acetone only. There were five treatments, and each treatment was replicated five times. The number of eggs laid on seeds of green gram was observed from 24 to 72 h. The percentage of oviposition inhibition was calculated by using the formula [45].

OI = [(NC − NT)/NC] ×100. where NT = No. of eggs in untreated and NT = No. of eggs laid in treated.

### 4.9. Detoxification Enzyme Inhibition of A. maritima Oil against Pulse Beetle

#### 4.9.1. Sample Preparation

Detoxification enzyme (Glutathione-S-Transferase and Acetylcholinesterae) inhibition activities were performed as per the standard methods [60,66,67]. Four different concentrations of *A. maritima* oil (4, 6, 8, and 10 mg/L for *C. chinensis* and 2, 4, 6, and 8 mg/L for *C. maculatus*) were chosen for detoxification enzyme inhibition activity based on fumigant toxicity assay described in Section 4.5. The adults who survived after 12 h of treatment (7–8 adults weighing 20 mg/concentration) were collected for enzyme assay. The adults in each test concentration were transferred to a centrifuge tube and homogenized in 0.1 M phosphate buffer (pH 7.4) in a ratio of 1:9. The weight of an adult (mg): the volume of buffer (mL) was kept in a ratio of 1:9. The adults were then homogenized with a homogenizer (Tarsons Micro Pestle). The homogenate was transferred immediately under ice bath conditions and then centrifuged at 12,000 rpm and 4 °C for 30 min. The supernatant was taken into a new centrifuge tube for protein estimation by Bradford assay [68] for all the concentrations before proceeding for enzyme assays. The same assay was repeated thrice for separate homogenates and then average values were taken for protein estimation.

#### 4.9.2. Protein Estimation

Protein estimation was done using the Bradford method [68] by adding 2 μL of homogenate, 38 μL of MilliQ to 160 μL of Bradford reagent in triplicates. After incubation of the mixture for 15 min at room temperature, the absorbance was measured at 595 nm. Absorbance was converted into protein concentrations and dilutions were made with respect to lower concentrations for the AChE assay.

#### 4.9.3. AChE Assay

The diluted 25 μL homogenates in triplicates were incubated for 30 min at room temperature with 25 μL of the reaction mixture (50 µL of DTNB, 50 μL of Acetothiocholine, 900 μL of assay buffer). The AChE activity was spectrophotometrically measured at 410 nm in a microplate reader (Biotek SYNERGY H1 Microplate Spectrophotometer). The enzyme activities were expressed as micromolar per milligram protein per minute (µmol/min/mg). For the determination of AChE, the Acetylcholinesterase Assay Kit was procured from Abcam, UK.

#### 4.9.4. GST Assay

The reaction contains 1000 μL of the solution, in which 75 μL of Assay buffer, 10 μL of the homogenized sample, 10 μL of glutathione were added. To initiate the reactions, 5 μL CDNB was added to each well in triplicates to microplate at room temperature. To measure the enzyme kinetics, a 96 well microplate was loaded with reaction solution and shaken for 10 s. After 60 s of lag time, the absorbance was read at 340 nm and the homogenates were read for 20 min at 37 °C with a microplate reader. The GST activity was determined from the extinction coefficient of 0.0096 µM^−1^ cm^−1^ for CDNB. The enzyme activities were expressed as micromolar per milligram protein per minute (µmol/min/mg protein). For the determination of GST and AChE enzyme, the Glutathione-S-Transferase Assay Kit was procured from Cayman Chemical, 1180 E, Ellsworth Road, Ann Arbor, MI, USA.

### 4.10. Statistical Analysis

The data on fumigant toxicity, ovipositional deterrent, and persistence of *A. maritima* oil was compiled. Lethal concentration (LC_50_), lethal time (LT_50_), and ovipositional deterrence (OD_50_) values were calculated by Probit analysis [69] using SPSS software v.16.0. The data on repellency, ovipositional deterrence, persistence, and enzyme inhibition were subjected to one-way ANOVA by SPSS software, and means were compared by Tukey’s post hoc test to know the significant differences between treatments. The assumptions of normality and homogeneity of variance test for different parameters/variables and no data transformations were required.

## 5. Conclusions

*A. maritima* oil showed promising fumigant toxicity (LC_50_ = 0.56 to 1.17 mg/L) against pulse beetle after 48 h of treatment. Higher concentrations of oil (6 and 8 mg/L) significantly inhibited the GST enzyme in *C. maculatus.* However, the EO of *A. maritima* may be recommended for the control of pulse beetle particularly grains stored in bins based on a safe waiting period, persistence studies, and economics.

## Figures and Tables

**Table 1:** Chemical composition of essential oil of *A. maritima*.

Sr. No.	Name	RI ^a^	RI ^b^	Area (%)	Mode of Identification
1	Santolina triene	908	903	2.18	MS, RI
2	Camphene	953	954	3.74	MS, RI
3	Sabinene	976	975	6.42	MS, RI
4	Myrcene	991	990	9.59	MS, RI
5	β-phellandrene	1031	1022	3.68	MS, RI
6	1,8-cineole	1033	1028	41.14	MS, RI
7	Terpinolene	1088	1090	2.50	MS, RI
8	*trans*-thujone	1112	1109	2.61	MS, RI
9	Chrysanthenyl acetate	1262	1259	0.96	MS, RI
10	Bornyl acetate	1284	1287	18.10	MS, RI
11	Sabinyl acetate	1291	1289	1.16	MS, RI
12	Isobornylpropanate	1381	1378	2.03	MS, RI
13	Germacrene-D	1480	1484	2.42	MS, RI
14	Isobornyl 2-Methyl butyrate	1520	1510	1.98	MS, RI
15	Unknown	-	-	1.50	MS, RI
	Total			98.51	
	Monoterpene hydrocarbons *			28.11	
	Oxygenated monoterpene *			45.73	
	Sesquiterpene hydrocarbons *			2.42	
	Oxygenated sesquiterpene *			0.0	

^a^ Retention index value of compounds in the literature (Adams 2007). ^b^ Retention index value determined relative to *n*-alkanes (C9–C24) on the DB-5 GC column. * Percentage of compounds class in analyzed essential oil samples.

**Table 2:** Fumigant toxicity of *A. maritima* oil against *C. chinensis* and *C. maculatus*.

	*C. chinensis*				
Time	LC_50_ (mg/L)	Confidence Limits (mg/L)	Slope ± SE	Chi-Square	*p*-Value
12 h	2.90	2.45–3.62	3.06 ± 0.57	2.83	0.42
24 h	2.06	1.72–2.45	3.08 ± 0.51	5.15	0.16
48 h	1.17	0.86–1.43	2.96 ± 0.51	2.95	0.40
	*C. maculatus*				
12 h	3.93	2.83–6.29	1.58 ± 0.29	2.51	0.47
24 h	1.91	0.96–3.63	0.87 ± 0.25	0.47	0.93
48 h	0.56	0.23–0.88	1.37 ± 0.31	4.42	0.22

**Table 3:** Residual toxicity of *C. chinensis* and *C. maculatus* adults fed *A. maritima* oil-treated grains stored for different periods.

Days after Treatment	Percent Mortality (24 and 48 h after Treatment)			
	*C. chinensis*		*C. maculatus*	
	24 h	48 h	24 h	48 h
10 DAT	50 ± 3.16 a	82 ± 2.00 a	28 ± 3.74 a	44 ± 4.00 a
20 DAT	6 ± 4.00 a	18 ± 3.74 b	20 ± 3.16 a	26 ± 4.00 b
30 DAT	4 ± 2.44 a	8 ± 2.00 c	4 ± 2.44 b	8 ± 3.74 c
40 DAT	0 ± 0.00 a	8 ± 2.00 c	4 ± 2.44 b	0 ± 0.00 c
F value	F_3,19_ = 54.07; *p* < 0.0001	F_3,19_ = 55.30; *p* < 0.0001	F_3,19_ = 16.00; *p* < 0.0001	F_3,19_ = 33.47; *p* < 0.0001

* Means followed by the same letters within a column do not differ significantly by Tukey’s HSD (*p* ≤ 0.05).

**Table 4:** LT_50_ values of *A. maritima* oil-treated grains (higher concentration) against adults of *C. chinensis* and *C. maculatus* at different storage periods of 10, 20, 30, and 40 days.

Time	LT_50_ (Days)	Confidence Limits (Days)	Slope ± SE	Chi-Square	*p*-Value
*C. chinensis*	14.49	12.79–16.11	6.26 ± 0.79	2.59	0.27
*C. maculatus*	9.33	5.88–11.96	2.48 ± 0.62	1.70	0.43

**Table 5:** Repellent activity of *A. maritima* oil against *C. chinensis* and *C. maculatus*.

Conc. (mg/L)	% Repellence (Hours after Treatment)				
	1 h	2 h	3 h	4 h	5 h
	*C. chinensis*				
1	24.00 ± 7.48 c	24.00 ± 4.00 d	16.00 ± 4.00 c	12.00 ± 4.90 c	12.00 ± 4.90 c
2	44.00 ± 7.48 c	44.00 ± 7.48 cd	40.00 ± 8.94 bc	28.00 ± 4.90 bc	30.00 ± 6.32 bc
4	56.00 ± 11.66 bc	56.00 ± 11.66 bc	52.00 ± 10.20 b	36.00 ± 16.00 bc	36.00 ± 7.48 bc
6	80.00 ± 6.32 ab	76.00 ± 4.00 ab	72.00 ± 8.00 ab	60.00 ± 14.14 ab	60.00 ± 14.14 ab
8	92.00 ± 4.90 a	88.00 ± 4.90 a	88.00 ± 4.90 a	88.00 ± 4.90 a	88.00 ± 4.90 a
	F_4,24_ = 11.97; *p* < 0.0001	F_4,24_ = 13.00; *p* < 0.0001	F_4,24_ = 13.56; *p* < 0.0001	F_4,24_ = 8.36; *p* < 0.0001	F_4,24_ = 12.60; *p* < 0.0001
	*C. maculatus*				
1	28.00 ± 8.00 c	20.00 ± 6.32 d	16.00 ± 4.00 d	16.00 ± 7.48 c	16.00 ± 4.00 c
2	40.00 ± 10.95 c	32.00 ± 4.90 cd	28.00 ± 8.00 cd	28.00 ± 4.90 bc	24.00 ± 4.00 bc
4	56.00 ± 9.80 bc	48.00 ± 4.90 bc	44.00 ± 4.00 bc	40.00 ± 6.32 bc	36.00 ± 7.48 bc
6	76.00 ± 7.48 ab	68.00 ± 4.90 b	56.00 ± 7.48 b	52.00 ± 10.20 b	48.00 ± 10.20 b
8	96.00 ± 4.00 a	92.00 ± 4.90 a	88.00 ± 4.90 a	88.00 ± 8.00 a	84.00 ± 7.48 a
	F_4,24_ = 10.61; *p* < 0.0001	F_4,24_ = 30.29; *p* < 0.0001	F_4,24_ = 21.95; *p* < 0.0001	F_4,24_ = 13.25; *p* < 0.0001	F_4,24_ = 14.29; *p* < 0.0001

* Mean of five replications; Means followed by the same letters within a column do not differ significantly by Tukey’s HSD (*p* ≤ 0.05).

**Table 6:** Ovipositional deterrence of *A. maritima* oil against *C. chinensis* and *C. maculatus* adults.

	*C. chinensis*				
Time (h)	OD_50_ (mg/L)	Confidence Limits (mg/L)	Slope ± SE	Chi-Square	*p*-Value
24 h	2.30	1.74–2.80	2.36 ± 0.27	3.59	0.31
48 h	3.09	2.50–3.66	2.28 ± 0.23	3.68	0.30
72 h	3.30	2.65–3.93	2.11 ± 0.21	1.83	0.61
	*C. maculatus*				
24 h	2.89	2.51–3.30	2.34 ± 0.19	4.42	0.22
48 h	3.36	2.85–3.90	1.94 ± 0.17	3.06	0.38
72 h	4.01	3.32–4.79	1.55 ± 0.15	4.80	0.19

## Supplementary Materials

The following are available online. Table S1: Repellent index of *A. maritima* oil against *C. chinensis* and *C. maculatus*. Table S2: Ovipositional inhibition of *A. maritima* oil against *C. chinensis*. Table S3: Ovipositional inhibition (deterrence) of *A. maritima* oil against *C. maculatus*. Table S4: Enzyme inhibition activity of *A. maritima* oil in *C. chinensis* and *C. maculatus* adults.

## Sample Availability

Not available.

## References

[1] Yadav D.N., Anand T., Sharma M., Gupta R.K. (2014). Microwave technology for disinfestation of cereals and pulses: An overview. J. Food Sci. Technol..

[2] Rajendran S., Pimentel D. (2002). Postharvest pest losses. Encyclopedia of Pest Management.

[3] Mogbo T.C., Akunne C.E., Ononye B.U. (2013). Evaluation of the efficacy of mixed leaf powders of *Vernonia amygdalina* (L.) and *Azadirachta indica* (A. Juss) against *Callosobruchus maculatus* (F.) (Coleoptera: Bruchidae). J. Biosci. Bioeng..

[4] Jayaram C.S., Chauhan N., Dolma S.K., Reddy S.G.E. (2022). Chemical composition and insecticidal activities of essential oils against the pulse beetle. Molecules.

[5] Sharma H.C., Gowda C.L.L., Stevenson P.C., Ridsdill-Smith T.J., Clement S.L., Rao G.V.R., Romeis J., Miles M., El-Bouhssini M. (2007). Host plant resistance and insect pest management in chickpea. Chickpea Breed. Manag..

[6] Varma S., Anadi P. (2010). Biology of pulse beetle (*Callosobruchus Chinensis* Linn., Coleoptera: Bruchidae) and their management through botanicals on stored mung grains in Allahabad region. Legume Res..

[7] Shaheen F.A., Khaliq A. (2005). Management of pulse beetle, *Callosobruchus chinensis* L. (Coleoptera: Bruchidae) in stored chickpea using ashes, red soil powder and turpentine oil. Pak. Entomol..

[8] Nenaah G.E., Ibrahim S.I. (2011). Chemical composition and the insecticidal activity of certain plants applied as powders and essential oils against two stored-products coleopteran beetles. J. Pest Sci..

[9] Mbata G.N., Payton M.E. (2013). Effect of monoterpenoids on oviposition and mortality of *Callosobruchus maculatus* (F.) (Coleoptera: Bruchidae) under hermetic conditions. J. Stored Prod. Res..

[10] Nenaah G.E. (2014). Chemical composition, toxicity and growth inhibitory activities of essential oils of three Achillea species and their nano-emulsions against *Tribolium castaneum* (Herbst). Ind. Crops Prod..

[11] Burt S. (2004). Essential oils: Their antibacterial properties and potential applications in foods-a review. Int. J. Food Microbiol..

[12] Kumar P., Mishra S., Malik A., Satya S. (2011). Insecticidal properties of Mentha species: A review. Ind. Crops Prod..

[13] Chauhan R.S., Kitchlu S., Ram G., Kaul M.K., Tava A. (2010). Chemical composition of capillene chemotype of *Artemisia dracunculus* L. from North-West Himalaya, India. Ind. Crops Prod..

[14] Abad M.J., Bedoya L.M., Apaza L., Bermejo P. (2012). The *Artemisia* L. genus: A review of bioactive essential oils. Molecules.

[15] Pandey V., Verma R.S., Chauhan A., Tiwari R. (2015). Compositional characteristics of the volatile oils of three *Artemisia* spp. from western Himalaya. J. Essent. Oil Res..

[16] Walia S., Rana A., Singh A., Sharma M., Reddy S.G.E., Kumar R. (2019). Influence of harvesting time on essential oil content, chemical composition and pesticidal activity of *Artemisia maritima* growing wild in the cold desert region of western Himalayas. J. Essent. Oil-Bear. Plants.

[17] Tan R.X., Zheng W.F., Tang H.Q. (1998). biologically active substances from the genus Artemisia. Planta Med..

[18] Li G., Yuan M., Li H., Deng C., Wang Q., Tang Y., Zhang H., Yu W., Xu Q., Zou Y. (2021). Safety and efficacy of artemisinin-piperaquine for treatment of COVID-19: An open-label, non-randomised and controlled trial. Int. J. Antimicrob. Agents.

[19] Tripathi A.K., Prajapati V., Aggarwal K.K., Khanuja S.P.S., Kumar S. (2000). Repellency and toxicity of oil from *Artemisia annua* to certain stored-product beetles. J. Econ. Entomol..

[20] Abd-Elhady H. (2012). Insecticidal activity and chemical composition of essential oil from *Artemisia Judaica* L. against *Callosobruchus maculatus* (F.) (*Coleoptera: Bruchidae*). J. Plant Prot. Res..

[21] Bozhüyük A.U., Kordali Ş., Kesdek M., Altınok M.A., Varcın M., Bozhüyük M.R. (2016). Insecticidal effects of essential oils obtained from six plants against *Callosobruchus maculatus* (F.) (Coleoptera: Bruchidae), a pest of cowpea (*Vigna unguiculata*) (L.). Fresenius Environ. Bull..

[22] Wang J., Zhu F., Zhou X.M., Niu C.Y., Lei C.L. (2006). Repellent and fumigant activity of essential oil from *Artemisia vulgaris* to *Tribolium castaneum* (Herbst) (Coleoptera: Tenebrionidae). J. Stored Prod. Res..

[23] Negahban M., Moharramipour S., Sefidkon F. (2006). Chemical composition and insecticidal activity of *Artemisia scoparia* essential oil against three coleopteran stored product insects. J. Asia-Pac. Entomol..

[24] Negahban M., Moharramipour S., Sefidkon F. (2007). Fumigant toxicity of essential oil from *Artemisia sieberi* Besser against three stored-product insects. J. Stored Prod. Res..

[25] Ahmed M., Peiwen Q., Gu Z., Liu Y., Sikandar A., Hussain D., Javeed A., Shafi J., Iqbal M.F., An R. (2020). Insecticidal activity and biochemical composition of *Citrullus colocynthis*, *Cannabis indica* and *Artemisia argyi* extracts against cabbage aphid (*Brevicoryne brassicae* L.). Sci. Rep..

[26] Huang X., Huang Y., Yang C., Liu T., Liu X., Yuan H. (2021). Isolation and insecticidal activity of essential oil from *Artemisia lavandulaefolia* DC. against *Plutella xylostella*. Toxins.

[27] Titouhi F., Amri M., Messaoud C., Haouel S., Youssfi S., Cherif A., Jemâa J.M.B. (2017). Protective effects of three Artemisia essential oils against *Callosobruchus maculatus* and *Bruchus rufimanus* (Coleoptera: Chrysomelidae) and the extended side-effects on their natural enemies. J. Stored Prod. Res..

[28] Mathela C.S., Kharkwal H., Shah G.C. (1994). Essential oil composition of some Himalayan Artemisia species. J. Essent. Oil Res..

[29] Bachrouch O., Ferjani N., Haouel S., Jemâa J.M.B. (2015). Major compounds and insecticidal activities of two Tunisian Artemisia essential oils toward two major coleopteran pests. Ind. Crops Prod..

[30] Mohan M., Pandey A.K., Singh P., Nautiyal M.K., Gupta S. (2016). Evaluation of *Artemisia maritima* L. essential oil for its chemical and biological properties against some foodborne pathogens. Anal. Chem. Lett..

[31] Sah S., Lohani H., Narayan O., Bartwal S., Chauhan N.K. (2010). Volatile constituents of *Artemisia maritima* Linn grown in Garhwal Himalaya. J. Essent. Oil-Bearing Plants.

[32] Stappen I., Wanner J., Tabanca N., Wedge D.E., Ali A., Khan I.A., Jirovetz L. (2014). Chemical composition and biological effects of *Artemisia maritima* and *Artemisia nilagirica* essential oils from wild plants of western Himalaya. Planta Med..

[33] Chaieb I., Ben Hamouda A., Tayeb W., Zarrad K., Bouslema T., Laarif A. (2018). The Tunisian Artemisia essential oil for reducing contamination of stored cereals by *Tribolium castaneum*. Food Technol. Biotechnol..

[34] Liu C.H., Mishra A.K., Tan R.X., Tang C., Yang H., Shen Y.F. (2006). Repellent and insecticidal activities of essential oils from *Artemisia princeps* and *Cinnamomum camphora* and their effect on seed germination of wheat and broad bean. Biores. Technol..

[35] Jaitak V., Singh B., Kaul V.K. (2008). Variability of volatile constituents in *Artemisia maritima* in western Himalaya. Nat. Prod. Res..

[36] Perry N.B., Anderson R.E., Brennan N.J., Douglas M.H., Heaney A.J., McGimpsey J.A., Smallfield B.M. (1999). Essential oils from Dalmatian sage (*Salvia officinalis* L.) variations among individuals, plant parts, seasons, and sites. J. Agric. Food Chem..

[37] Abou-Taleb H.K., Mohamed M.I., Shawir M.S., Abdelgaleil S.A. (2016). Insecticidal properties of essential oils against *Tribolium castaneum* (Herbst) and their inhibitory effects on acetylcholinesterase and adenosine triphosphatases. Nat. Prod. Res..

[38] Zhang Z., Guo S.S., Zhang W.J., Geng Z.F., Liang J.Y., Dua S.S., Wang C.F., Deng Z.W. (2017). Essential oil and polyacetylenes from *Artemisia ordosica* and their bioactivities against *Tribolium castaneum* Herbst (Coleoptera: Tenebrionidae). Ind. Crops Prod..

[39] Goel D., Goel R., Singh V. (2007). Composition of the essential oil from the root of *Artemisia annua*. J. Nat. Med..

[40] Lee S.E., Lee B.H., Choi W.S., Park B.S., Kim J.G., Campbell B.C. (2001). Fumigant toxicity of volatile natural products from Korean spices and medicinal plants towards the rice weevil, *Sitophilus oryzae* (L.). Pest Manag. Sci..

[41] Tripathi A.K., Prajapati V., Aggarwal K.K., Kumar S. (2001). Toxicity, feeding deterrence, and effect of activity of 1,8-cineole from *Artemisia annua* on progeny production of *Tribolium castanaeum* (Coleoptera: Tenebrionidae). J. Econ. Entomol..

[42] Ogendo J.O., Kostyukovsky M., Ravid U., Matasyoh J.C., Deng A.L., Omolo E.O., Kariuki S.T., Shaaya E. (2008). Bioactivity of *Ocimum gratissimum* L. oil and two of its constituents against five insect pests attacking stored food products. J. Stored Prod. Res..

[43] Shukla R., Singh P., Prakash B., Kumar A., Mishra P.K., Dubey N.K. (2011). Efficacy of essential oils of *Lippia alba* (Mill.) N.E. Brown and *Callistemon lanceolatus* (Sm.) Sweet and their major constituents on mortality, oviposition and feeding behaviour of pulse beetle, *Callosobruchus chinensis* L.. J. Sci. Food Agric..

[44] Chiluwal K., Kim J., Bae S.D., Park C.G. (2017). Essential oils from selected wooden species and their major components as repellents and oviposition deterrents of *Callosobruchus chinensis* (L.). J. Asia Pac. Entomol..

[45] Isman M.B. (2000). Plant essential oils for pest and disease management. Crop Prot..

[46] Bakkali F., Averbeck S., Averbeck D., Idaomar M. (2008). Biological effects of essential oils: A review. Food Chem. Toxicol..

[47] Coloma A.G., Reina M., Diaz C.E., Fraga B.M. (2010). Natural product-based biopesticides for insect. In comprehensive natural products II. Chem. Biol..

[48] Paolini J., El Ouariachi E.M., Bouyanzer A., Hammouti B., Desjobert J.M., Costa J., Muselli A. (2010). Chemical variability of *Artemisia herba-alba* Asso essential oils from East Morocco. Chem. Papers.

[49] Sharifian I., Hashemi S.M., Aghali M., Alizadeh M. (2012). Insecticidal activity ofessential oil of *Artemisia herba alba* against three stored product beetles. Biharean Biol..

[50] Liu X.C., Li Y., Wang T., Wang Q., Liu Z.L. (2014). Chemical composition and insecticidal activity of essential oil of *Artemisia frigida* Willd (Compositae) against two grain storage insects. Trop. J. Pharm. Res..

[51] Russell R.J., Scott C., Jackson C.J., Pandey R., Pandey G., Taylor M.C., Oakeshott J.G. (2011). The evolution of new enzyme function: Lessons from xenobiotic metabolizing bacteria versus insecticide-resistant insects. Evol. App..

[52] Ramsey J.S., Rider D.S., Walsh T.K., De Vos M., Gordon K.H.J., Ponnala L., Jander G. (2010). Comparative analysis of detoxification enzymes in *Acyrthosiphon pisum* and *Myzus persicae*. Insect Mol. Biol..

[53] Li X.C., Schuler M.A., Berenbaum M.R. (2007). Molecular mechanisms of metabolic resistance to synthetic and natural xenobiotics. Annu. Rev. Entomol..

[54] Yu S.J., Hsu E.L. (1993). Induction of detoxification enzymes in phytophagous insects: Role of insecticide synergists, larval age, and species. Arch. Insect Biochem. Physiol..

[55] Bouayad N., Rharrabe K., Ghailani N.N., Jbilou R., Domínguez P.C. (2013). Insecticidal effects of Moroccan plant extracts on development, energy reserves and enzymatic activities of *Plodia interpunctella*. Span. J. Agric. Res..

[56] Clark A.G., Shamaan N.A., Sinclair M.D., Dauterman W.C. (1986). Insecticide metabolism by multiple glutathione S-transferases in two strains of the house fly, *Musca domestica* (L.). Pestic. Biochem. Physiol..

[57] Hu Z.D., Xia F.E.N.G., Lin Q.S., Chen H.Y., Li Z.Y., Fei Y.I.N., Liang P., Gao X.W. (2014). Biochemical mechanism of chlorantraniliprole resistance in the diamondback moth, *Plutella xylostella* Linnaeus. J. Integr. Agric..

[58] Tak J.H., Isman M.B. (2016). Metabolism of citral, the major constituent of lemongrass oil, in the cabbage looper, *Trichoplusia ni*, and effects of enzyme inhibitors on toxicity and metabolism. Pestic. Biochem. Physiol..

[59] Gao X.W. (2012). Insect Adaptation to Plant Allele Chemicals Based on Detoxification: Helicoverpa armigera as an Example.

[60] Yang H., Piao X., Zhang L., Song S., Xu Y. (2018). Ginsenosides from the stems and leaves of Panax ginseng show antifeedant activity against *Plutella xylostella* (Linnaeus). Ind. Crops Prod..

[61] Hu J., Wang W., Dai J., Zhu L. (2019). Chemical composition and biological activity against *Tribolium castaneum* (Coleoptera: Tenebrionidae) of *Artemisia brachyloba* essential oil. Ind. Crops Prod..

[62] Koundal R., Reddy S.G.E., Dolma S.K., Singh B. (2016). Chemical composition and insecticidal activities of essential oils against diamondback moth, *Plutella xylostella* (L.) (Lepidoptera: Yponomeutidae). Nat. Prod. Res..

[63] Eccles K., George Y.L.P., Mohammed F.K., Khan A. (2019). Efficacy of *Artocarpus altilis* (Parkinson) Fosberg extracts on contact mortality, repellency, oviposition deterrency and fumigant toxicity of *Callosobruchus maculatus* (F.) (Coleoptera: Bruchidae). Int. J. Pest Manag..

[64] Nerio L.S., Olivero-Verbel J., Stashenko E.E. (2009). Repellent activity of essential oils from seven aromatic plants grown in Colombia against *Sitophilus zeamais* Motschulsky (Coleoptera). J. Stored Prod. Res..

[65] Kogan M., Goeden R.D. (1970). The Host-Plant Range of *Lema trilineatadaturaphila* (Coleoptera: Chrysomelidae). Ann. Entomol. Soc. Am..

[66] Ellman G.L., Courtney K.D., Andres V., Featherstone R.M. (1961). A new and rapid colorimetric determination of acetylcholinesterase activity. Biochem. Pharmacol..

[67] Larson R.T., Lorch J.M., Pridgeon J.W., Becnel J.J., Clark G.G. (2010). The biological activity of α-Mangostin, a larvicidal botanic mosquito sterol carrier protein-2 inhibitor. J. Med. Entomol..

[68] Bradford M.M. (1976). A rapid and sensitive method for the quantitation of microgram quantities of protein utilizing the principle of protein-dye binding. Anal. Biochem..

[69] Finney D.J. (1971). Probit Analysis.

