# Supplementary material for: Chemical Composition, Insecticidal, Persistence and Detoxification Enzyme Inhibition Activities of Essential Oil of Artemisia maritima against the Pulse Beetle
Source: Molecules. 2022 Feb 25;27(5):1547. doi: 10.3390/molecules27051547 (PMC8911588; doi:10.3390/molecules27051547)
Supplement: Supplementary file 1 [file molecules-27-01547-s001.zip › molecules-1591772-supplementary.pdf]

Supplementary material

Chemical Composition, Insecticidal, Persistence and Detoxification Enzyme Inhibition Activities of Essential Oil of *Artemisia maritima* against the Pulse Beetle

Nandita Chauhan [1], Urvashi Kashyap [1,2], Shudh Kirti Dolma [1,2] and Sajjalavarahalli G. Eswara Reddy [1,2,*]

[1] Entomology Laboratory, Agrotechnology Division, CSIR-Institute of Himalayan Bioresource Technology, Palampur 176061, India; nanditachauhan796@gmail.com (N.C.); kashurvi5991@gmail.com (U.K.); skdolma@gmail.com (S.K.D.)

[2] Academy of Scientific and Innovative Research (AcSIR), Ghaziabad 201002, India

* Correspondence: ereddy@ihbt.res.in or ereddy2001@yahoo.com

Table S1. Repellent index of *A. maritima* oil against *C. chinensis* and *C. maculatus*

Conc. (mg/L)	Conc. (mg/L)	Repellent Index (RI) (Hours after treatment (*Mean ± SD)				
		1 h	2 h	3 h	4 h	5 h
C. chinensis	8	0.12 ± 0.05 (I)	0.20 ± 0.06 (I)	0.24 ± 0.07 (I)	0.32 ± 0.08 (I)	0.32 ± 0.08 (I)
	6	0.20 ± 0.06 (I)	0.24 ± 0.04 (I)	0.24 ± 0.07 (I)	0.40 ± 0.14 (I)	0.40 ± 0.14 (I)
	4	0.44 ± 0.12 (I)	0.44 ± 0.02 (I)	0.48 ± 0.10 (I)	0.64 ± 0.16 (I)	0.68 ± 0.19 (I)
	2	0.56 ± 0.07 (I)	0.56 ± 0.07 (I)	0.56 ± 0.12 (I)	0.72 ± 0.14 (I)	0.72 ± 0.14 (I)
	1	0.80 ± 0.14 (I)	0.96 ± 0.07 (I)	1.12 ± 0.10 (I)	1.08 ± 0.05 (I)	1.08 ± 0.05 (I)
C. maculatus	8	0.16 ± 0.75 (I)	0.04 ± 0.04 (I)	0.08 ± 0.05 (I)	0.04 ± 0.04 (I)	0.08 ± 0.05 (I)
	6	0.28 ± 0.10 (I)	0.24 ± 0.07 (I)	0.32 ± 0.10 (I)	0.32 ± 0.14 (I)	0.20 ± 0.11 (I)
	4	0.48 ± 0.10 (I)	0.44 ± 0.07 (I)	0.56 ± 0.04 (I)	0.48 ± 0.14 (I)	0.44 ± 0.07 (I)
	2	0.64 ± 0.13 (I)	0.64 ± 0.07 (I)	0.68 ± 0.10 (I)	0.60 ± 0.06 (I)	0.60 ± 0.06 (I)
	1	0.80 ± 0.15 (I)	0.72 ± 0.17 (I)	0.76 ± 0.16 (I)	0.92 ± 0.10 (I)	0.92 ± 0.80 (I)

*Mean of five replications; R- Repellent (RI less than 1-SD), I-Indifferent (RI in between 1-SD and 1+SD), A-Attractant (RI greater than 1+ SD).

Table S2. Ovipositional inhibition of *A. maritima* oil against *C. chinensis*

Conc. (mg/L)	Per cent ovipositional deterrence		
	24 h	48 h	72 h
12	100.0 ± 0.0 a	100.0 ± 0.0 a	98.28 ± 1.05 a
8	94.38 ± 2.66 a	92.34 ± 2.72 a	93.28 ± 1.48 a
4	78.20 ± 3.12 ab	74.70 ± 3.29 b	76.12 ± 2.65 b
2	67.38 ± 2.89 b	64.08 ± 3.89 b	67.40 ± 2.72 b
1	41.06 ± 12.12 c	36.40 ± 6.27 c	47.76 ± 4.38 c
	$F_{4, 24}$=16.08; p<0.0001	$F_{4, 24}$=43.43; p<0.0001	$F_{4, 24}$=56.45; p<0.0001

*Mean of three replications; Means followed by the same letters within a column do not differ significantly by Tukey's HSD ($P \leq 0.05$)

Table S3. Ovipositional inhibition (deterrence) of *A. maritima* oil against *C. maculatus*

Conc. (mg/L)	Per cent ovipositional deterrence		
	24 h	48 h	72 h
8	100.0 ± 0.0 a	91.28 ± 3.40 a	81.78 ± 3.79 a
4	86.54 ± 6.58 ab	81.70 ± 4.91 a	74.36 ± 2.90 a
2	70.00 ± 5.46 b	68.64 ± 4.68 b	69.40 ± 2.37 a
1	41.16 ± 6.92 c	42.70 ± 8.71 b	48.04 ± 5.58 b
0.5	23.06 ± 6.72 c	17.02 ± 3.45 c	22.18 ± 3.23 c
	$F_{4, 24}$=30.38; p<0.0001	$F_{4, 24}$=31.55; p<0.0001	$F_{4, 24}$=41.78; p<0.0001

*Mean of three replications; Means followed by the same letters within a column do not differ significantly by Tukey's HSD ($p \leq 0.05$)

Table S4. Enzyme inhibition activity of *A. maritima* oil in *C. chinensis and C. maculatus* adults

Conc. (mg/L)	*C. chinensis*	
	AchE	GST
10	13.96 ± 2.46 a	12.38± 1.18 a
8	11.46 ± 0.24 a	13.41± 0.92 a
6	9.16 ± 0.91 ab	14.96± 0.92 a
4	9.98 ± 0.48 ab	16.51± 1.85 a
Control	5.56 ± 0.061 b	21.92± 3.90 a
	$F_{4,14}$=6.62; p >0.007	$F_{4,14}$=3.21; p >0.061
Conc. (mg/L)	*C. maculatus*	
8	19.53 ± 1.73 a	5.61± 0.51a
6	18.571 ± 3.02 a	7.14 ± 1.02 ab
4	14.61 ± 0.27 ab	12.23 ± 2.33 bc
2	9.49 ± 0.47 b	15.29 ± 1.53 c
Control	10.21 ± 0.83 b	18.35 ± 0.88 c
	$F_{4,14}$=8.14; p <0.003	$F_{4,14}$=14.60; p <0.0001

*Mean of three replications; Means followed by the same letters within a column do not differ significantly by Tukey's HSD ($p \leq 0.05$)
